# Thrombotic and Thromboembolic Complications After Vaccination Against COVID-19: A Systematic Review

**DOI:** 10.7759/cureus.37275

**Published:** 2023-04-07

**Authors:** TT Favas, Neha Lall, Deepika Joshi, Varun K Singh, Abhishek Pathak, Kamalesh Chakravarty, Vijaya Nath Mishra, Rameshwar N Chaurasia, Anand Kumar

**Affiliations:** 1 Department of Neurology, Institute of Medical Science, Banaras Hindu University, Varanasi, IND; 2 Department of Radiation Oncology, Mahamana Pandit Madan Mohan Malaviya Cancer Centre, Varanasi, IND; 3 Department of Neurology, Postgraduate Institute of Medical Education and Research, Chandigarh, IND; 4 Department of Neurology, Institute of Medical Science, Banaras Hindu University, varanasi, IND

**Keywords:** venous thrombosis, vaccine-induced prothrombotic immune thrombocytopenia, thrombosis with thrombocytopenia syndrome, thrombosis, covid-19 vaccines

## Abstract

Thromboembolic complications after the COVID-19 vaccination have been reported from all over the world. We aimed to identify the thrombotic and thromboembolic complications that can arise after receiving various types of COVID-19 vaccines, their frequency, and distinguishing characteristics. Articles published in Medline/PubMed, Scopus, EMBASE, Google Scholar, EBSCO, Web of Science, the Cochrane Library, the CDC database, the WHO database, ClinicalTrials.gov, and servers like medRxiv.org and bioRxiv.org, as well as the websites of several reporting authorities between December 1, 2019, and July 29, 2021, were searched. Studies were included if they reported any thromboembolic complications post-COVID-19 vaccination and excluded editorials, systematic reviews, meta-analyses, narrative reviews, and commentaries. Two reviewers independently extracted the data and conducted the quality assessment. Thromboembolic events and associated hemorrhagic complications after various types of COVID-19 vaccines, their frequency, and distinguishing characteristics were assessed. The protocol was registered at PROSPERO (ID-CRD42021257862). There were 59 articles, enrolling 202 patients. We also studied data from two nationwide registries and surveillance. The mean age of presentation was 47 ± 15.5 (mean ± SD) years, and 71.1% of the reported cases were females. The majority of events were with the AstraZeneca vaccine and with the first dose. Of these, 74.8% were venous thromboembolic events, 12.7% were arterial thromboembolic events, and the rest were hemorrhagic complications. The most common reported event was cerebral venous sinus thrombosis (65.8%), followed by pulmonary embolism, splanchnic vein thrombosis, deep vein thrombosis, and ischemic and hemorrhagic stroke. The majority had thrombocytopenia, high D-dimer, and anti-PF4 antibodies. The case fatality rate was 26.5%. In our study, 26/59 of the papers were of fair quality. The data from two nationwide registries and surveillance revealed 6347 venous and arterial thromboembolic events post-COVID-19 vaccinations. COVID-19 vaccinations have been linked to thrombotic and thromboembolic complications. However, the benefits far outweigh the risks. Clinicians should be aware of these complications because they may be fatal and because prompt identification and treatment can prevent fatalities.

## Introduction and background

COVID-19 is caused by infection from the severe acute respiratory syndrome coronavirus 2 (SARS-CoV-2), and the first case was reported from Wuhan, Hubei Province, China, in December 2019 [1]. COVID-19 was declared a pandemic by the World Health Organization (WHO) on March 11, 2020 [1]. It is now evident that COVID-19 is associated with prothrombotic complications due to immunothrombosis and endothelial dysfunction. Immunothrombosis is caused by the activation of neutrophils and monocytes and causes dysregulated coagulation cascade activation. This in turn leads to the formation of thrombi in blood vessels [2].

One way to combat the SARS-CoV-2 pandemic is to develop and distribute vaccines as quickly as possible. The WHO listed various vaccines for emergency use, and the first vaccine listed for WHO emergency use listing (EUL) was the Pfizer/BioNTech vaccine (BNT162b2) on December 31, 2020 [3]. WHO also listed two versions of the AstraZeneca/Oxford vaccine developed by AstraZeneca/Oxford - the Serum Institute of India/Covishield and AstraZeneca/AZD1222 vaccines (ChAdOx1 nCov-19) on 15 February 2021, produced by the Serum Institute of India (SII) and AstraZeneca-SKBio (Republic of Korea) respectively; the Janssen/Ad26.COV 2.S vaccine developed by Johnson & Johnson/Janssen (J&J/Janssen) on 12 March 2021; the Moderna vaccine (mRNA 1273) on 30 April 2021; the Sinopharm COVID-19 vaccine produced by Beijing Bio-Institute of Biological Products Co Ltd, a subsidiary of China National Biotec Group on 07 May 2021; and the Sinovac-CoronaVac on 01 June 2021 [3]. Pfizer/BioNTech and Moderna are messenger RNA-based vaccines that encode the spike protein antigen of SARS-CoV-2; AstraZeneca is a recombinant chimpanzee adenoviral vector vaccine that encodes the spike glycoprotein of SARS-CoV-2; and the J&J/Janssen vaccine is a recombinant adenovirus type 26 vector vaccine that encodes the SARS-CoV-2 spike glycoprotein [3]. According to WHO data, globally as of September 5, 2021, a total of 5,352,927,296 vaccine doses of 13 different types of vaccines have been administered, with 2,018,604,130 people receiving at least one dose and 1,241,236,974 people receiving a full dose of vaccine [4].

Concerns about possible thrombotic problems following vaccination with the AstraZeneca vaccine were raised after reports from the European Medicines Agency (EMA) on March 10, 2021 [5]. Following the report, various European governments temporarily suspended the use of the AstraZeneca vaccine, although there was no convincing evidence that vaccination caused these problems [6]. The EMA's safety committee, the Pharmacovigilance Risk Assessment Committee (PRAC), concluded on March 18th that the benefits outweighed the possible risks in combating the COVID-19 outbreak, that there is no overall risk of thromboembolic events, and that the AstraZeneca vaccine could be used while further investigation and assessment are ongoing [7]. A Joint Centers for Disease Control and Prevention (CDC) and US Food and Drug Administration (FDA) Statement on the J&J/Janssen vaccine was released on April 13th, 2021, citing six cases of cerebral venous sinus thrombosis (CVST) after receiving the J&J/Janssen vaccine in the United States and recommending that the vaccine be halted until the next review [8]. The J&J/Janssen vaccine was reintroduced in the United States on April 23, 2021 [8]. Multiple cases of rare thrombotic events such as CVST have been observed around the world after receiving COVID-19 adenovirus-based vaccines [9,10]. The original designation used to describe COVID-19 vaccine-associated thrombosis with thrombocytopenia was “vaccine-induced prothrombotic immune thrombocytopenia” (VIPIT) [11]. Other terms used include vaccine-induced immune thrombotic thrombocytopenia (VITT) and vaccine-associated (immune) thrombotic thrombocytopenia (VATT) [11]. Most reporting agencies now use the term “thrombosis with thrombocytopenia syndrome” (TTS) [9,11,12]. The purpose of this systematic review is to identify the thrombotic and thromboembolic complications that can arise after receiving various types of COVID-19 vaccines, their frequency and distinguishing characteristics, as well as the vaccine dose, timing of onset of symptoms after vaccination, clinical features, laboratory and imaging results, treatment received, and outcome.

## Review

Methods

Selection Criteria and Search Strategy

We searched the following electronic databases for articles published between December 1, 2019, and July 29, 2021: Medline, PubMed, Scopus, EMBASE, Google Scholar, EBSCO, Web of Science, Cochrane Library, CDC database, WHO database, ClinicalTrials.gov, and servers like medRxiv.org and bioRxiv.org. We also looked at the websites of several reporting authorities, including the Vaccine Adverse Event Reporting System (VAERS), the Medicines and Healthcare Products Regulatory Agency (MHRA), and the Ministry of Health and Family Welfare of India (MOHFW). Relevant MeSH terms and keywords were used with an additional filter of “studies in human subjects” (Table 1). There were no language restrictions. A primary search was conducted between June 6, 2021, and July 15, 2021, and a final search was conducted on July 29, 2021. To ensure literature saturation, we reviewed the references of all studies included. In May 2021, the protocol for this review was prospectively registered at PROSPERO (ID-CRD42021257862).

**Table 1:** MeSH terms and keywords used

MeSH terms and keywords used			
“COVID-19 vaccine” “COVID-19 vaccination” “ChAdOx1 nCoV-19 Vaccination” “ChAdOx1” “ChAdOx1 nCoV-19 adenoviral vector vaccine” “Adenoviral vector vaccine” “AstraZeneca vaccine” “Vaxzevria vaccine” “AZD1222” “Oxford vaccine” “messenger RNA (mRNA)–based vaccines” “BNT162b2” “mRNA Covid-19 vaccine” “Pfizer–BioNTechvaccine” “Pfizer vaccine” “Pfizer covid-19 vaccine” “mRNA-1273 vaccine” “Moderna vaccine” “Ad26.COV2.S” “JNJ-78436735” “Johnson & Johnson/Janssen vaccine” “Johnson Johnson vaccine” “Johnson & Johnson vaccine” “J&J vaccine”	“SARS-CoV-2 spike glycoprotein” “recombinant adenoviral vector vaccine” “Anhui Zhifei Longcom” “RBD-dimer vaccine” “ZF2001 vaccine” “Bharat Biotech” “COVAXIN vaccine” “Serum Institute of India vaccine” “Covishield vaccine” “Gamelaya” “Sputnik vaccine” “Sputnik V vaccine” “Gam-COVID-Vac vaccine” “BBV152 vaccine” “Ad5-nCoV vaccine” “Cansino” “KoviVac vaccine” “Chumakov Center” “EpiVacCorona vaccine” “FBRI” “Sinopharm vaccine” “BBIBP-CorV vaccine” “CoronaVac vaccine”	“Sinovac” “Kazakhstan vaccine” “QazVac vaccine” “QazCovid-in” “Kazakhstan RIBSP” “COVID-19 treatment” “COVID 19 vaccine” “SARS-CoV-2 vaccine” “2019 novel coronavirus vaccine” “2019-nCOV vaccine” “Severe acute respiratory syndrome coronavirus 2 vaccine” “Wuhan coronavirus vaccine” “Coronavirus disease 2019 vaccine” “thrombosis” “Thrombotic complications” “thromboembolic” “Cerebral venous sinus thrombosis” “Cerebral thrombosis” “Dural sinus thrombosis” “cvst” “cvt” "dvt" “Deep vein thrombosis” “Portal vein thrombosis”	“myocardial infarction” “heart attack” “mesenteric thrombosis” "venous thrombosis" "arterial thrombosis" "thrombocytopenia" “thrombotic thrombocytopenia” "venous sinus thrombosis" “anti-PF4” “PF4” “anti-platelet factor 4” “prothrombotic thrombocytopenia” “autoimmune thrombocytopenia” “immune-mediated thrombocytopenia” “vaccine-induced HIT” “vaccine-induced prothrombotic immune thrombocytopenia” “vaccine-induced immune thrombotic thrombocytopenia” “VIPIT” “VITT” “vaccine-induced thrombocytopenia and bleeding” “thrombotic and thrombocytopenia syndrome” “TTS” “immune thrombocytopenia and bleeding”

Inclusion and Exclusion Criteria

All case-control studies, cohort studies, retrospective studies, case series, and case reports were included. There were no randomized controlled trials or non-randomized controlled trials. We also incorporated data from various reporting agencies and analyzed it separately. Studies were included if they met the following criteria: (1) use of any type of vaccination for COVID-19; (2) studies that reported thrombosis, thrombocytopenia, or sudden cardiac death as a complication; and (3) no other obvious or confirmed cause for thrombotic complications. Editorials, systematic reviews, meta-analyses, narrative reviews, full texts not available, and commentaries were excluded. Animal and post-mortem studies, as well as those that could not be translated into English, were removed. The same cases can be reported in different databases; however, we made an effort to prevent duplication by comparing the patient's age, sex, date of admission, hospital name, and clinical, laboratory, and radiological characteristics. Additionally, we emailed the relevant corresponding author for clarification if there were any uncertainties.

Data Extraction and Study Quality Assessment

Two team members (AK and TF) searched and extracted data from various databases. Two reviewers independently reviewed all the articles and selected articles based on inclusion and exclusion criteria. Duplicate studies were excluded from the analysis. Any discrepancies were resolved by discussing them with the third reviewer. Reporting was done according to the recommendations of the Preferred Reporting Items for Systematic Reviews and Meta-Analyses statement (PRISMA). Two investigators (AK and TF) independently assessed the risk of bias in the included studies with the National Institutes of Health (NIH) Quality Assessment Tool [13]. The following information was extracted: author's name, publication date, study design, sample size, demographic characteristics, age, gender, comorbidities, type of vaccine received, number of doses received, time of onset of symptoms after vaccination, site of thrombosis and incidence, clinical features, laboratory parameters, imaging features, treatment received, patient outcome, study follow-up, and limitations.

Outcome Measures and Data Synthesis Strategy

We assessed the venous and arterial thromboembolic manifestations reported following different types of vaccination against COVID-19, as well as the vaccine dose, timing of onset of symptoms after vaccination, clinical features, laboratory and imaging results, treatment received, and outcome. Because there were few registries available and to prevent duplication with the reported cases, the descriptive analysis did not include national registries, surveillance, or reporting agencies' data. For the categorical variables, we used simple and relative frequencies and proportions. We employed measures of central tendency (mean or median) and dispersion (standard error and standard deviation) for continuous variables. 

Results

**Figure 1:**
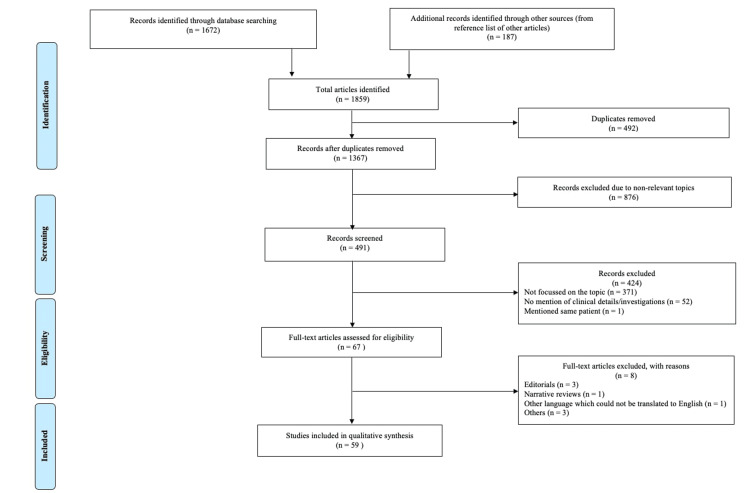
PRISMA flow chart for study selection

Among the 1859 articles identified through database searching and other sources, 59 studies enrolling 202 patients were included in this systematic review (Figure 1).

Out of them, one was a retrospective cohort, one was a retrospective survey, 17 were case series (Table 2), and the rest were case reports. Most of the studies reported were from the United States, the United Kingdom, and Germany. A population-based cohort study and a nested incident-matched case-control study were also included. These two investigations reported a total of 6347 venous and arterial thromboembolic events. We also looked at three independent registries from the United States, the United Kingdom, and India.

**Table 2:** Characteristics of case series and observational studies included Abbreviations: CVST, cerebral venous sinus thrombosis; DVT, deep vein thrombosis; DIC, disseminated intravascular coagulation; IVIG, intravenous immunoglobulin; LMWH, low molecular weight heparin; HTN, hypertension; DM, diabetes mellitus; SAH, subarachnoid haemorrhage; MCA, middle cerebral artery; UFH, unfractionated Heparin; TIA, transient ischemic attack; ICA, internal carotid artery; PE, plasmapheresis/plasma exchange; CT, Computed tomography; NA, not available N=total number of patients

First Author	Published journal, date of publication	Study design, study setting	Study population	Total number of patients (n) with venous or arterial events	Sex female, n (%)	Age (years), mean ± SD or median (range) or median (IQR)	COVID-19 tests	Vaccine type, dose	Time period from vaccination to first symptoms (days)	Diagnosis	Comorbidities and risk factors	Blood investigations	Antiplatelet antibodies and platelet-activation assay	Previous exposure to heparin	Treatment received	Outcome
Wolf ME [14]	Journal of Clinical Medicine, 09 April 2021	Case series, Germany	Patients treated by authors	3	3 (100)	22, 46, 36	SARS-CoV-2-PCR -negative in all	AstraZeneca, all first dose	4, 8, 7	CVST (1); CVST with haemorrhage (2)	No	Thrombocytopenia (3); Elevated D-dimer (3); Thrombophilia panel negative (3)	Anti-PF4 antibodies – positive in all	NA	Endovascular rheolysis (3); Enoxaparin (3); Dabigatran (3); Danaparoid (2)	Improved (3)
Greinacher A [15]	The New England Journal of Medicine, 09 April 2021	Case series, Germany and Austria	Patients in whom clinical data available by March 15, 2021	11	9 (82)	Median age – 36 (range, 22-49)	NA	AstraZeneca, first dose in one and others, NA	Range, 5-16	CVST (9); Splanchnic vein thrombosis (3); Pulmonary embolism (3); Aortoiliac thrombus (1); Cerebral haemorrhage (1); DIC (5); Multiple (5); Others (1)	Pre-existing von Willebrand disease, anticardiolipin antibod­ies, and factor V Leiden (1); Chronic neurologic disease (1); Oral contraceptive use (1); Hormonal intrauterine device (2); NA (1)	Thrombocytopenia (11)	Anti-PF4 antibodies - positive in 9/9; PF4-dependent platelet-activation assay - positive in 9/9	No	Heparin (5); No treatment (2); NA (2); apixaban (1)	Died (6); Recovering (4); Unknown (1)
Schultz NH [16]	The New England Journal of Medicine, 09 April 2021	Brief report, Norway	Health care workers	5	4 (80)	Median age – 39 (range, 32-54)	SARS-CoV-2 antibody test: nucleocapsid protein negative in all and spike protein positive in all	AstraZeneca, all dose	Range, 7 - 10	CVST with haemorrhage (4); Splanchnic vein and abasivertebral veins thrombosis (1)	HTN, Hormone-replacement Therapy (1); Contraceptive pill (1); Contraceptive vaginal ring (1)	Thrombocytopenia (5); Elevated D-dimer (5)	Anti-PF4 antibodies – positive in all	No	Platelet transfusions (4); Steroids (4); IVIG (4); LMWH (4); Heparin (1)	Died (3); Full recovery (2)
Scully M [17]	The New England Journal of Medicine, 16 April 2021	Case series, United Kingdom	Patients referred	23	14 (61)	Median age - 46 (range, 21-77)	SARS-CoV-2-PCR -negative in all	AstraZeneca, all first dose	Range, 6 - 24	CVST (11); CVST with intracerebral haemorrhage (2); Pulmonary embolism (5); Splanchnic vein thrombosis (3); DVT (2); Multiple (7); SAH (1); Others (2); Bilateral adrenal haemorrhage (1); Ischemic stroke (MCA territory) (2); Acute myocardial infarction (1); Aortic thrombosis (1); Cerebral haemorrhage (1)	History of DVT (1); Combined oral contraceptive use (1)	Thrombocytopenia (22/22); Elevated D-dimer (21/21); Low fibrinogen levels (13); Lupus anticoagulant - positive (5/10); Anticardiolipin antibodies and anti–β2- glycoprotein 1b antibodies – negative (5/10)	Anti-PF4 antibodies -positive in 22	No	NA	Died (7)
Mehta PR [18]	Brain, Behavior, and Immunity, 20 April 2021	Case series, United Kingdom	Cases presented early during the vaccination programme	2	0 (0) (Both male)	32, 25	SARS-CoV-2-PCR -negative	AstraZeneca, both first dose	9, 6	CVST with intracerebral haemorrhage and SAH (2)	Ex-smoker (1); Smoker (1); Primary sclerosing cholangitis and migraine (1)	Thrombocytopenia (2); Low fibrinogen levels (2); Factor V Leiden - heterozygous for the c.1601G > A (p. Arg534Gln) variant in one	Anti-PF4 antibodies -positive in 1/1	NA	UFH (1); Platelet (1; Steroids (1); IVIG (1); Symptomatic (1)	Died (2)
Haakonsen HB [19]	The Journal of the Norwegian Medical Association, 28 April 2021	Short case report, Norway	Health care professional	2	1 (50)	30s, 40s	NA	AstraZeneca, NA	27, 29	DVT (2)	Hypothyroidism (1)	Platelets and D-dimer normal	Anti-PF4 antibodies -negative in 1/1	NA	Apixaban (1); Rivaroxaban (1)	NA
Tiede A [20]	Blood, 28 April 2021	Brief report, Germany	Consecutive single-center cohort, between 8 March and 4 April 2021	5	5 (100)	41, 67, 63, 61, 61	SARS-CoV-2-PCR -negative in all	AstraZeneca, all first dose	Range, 5 - 11	CVST with haemorrhage and thrombotic microangiopathy (1); TIA (1); Splanchnic vein thrombosis (1); Ischemic stroke with haemorrhagic transformation (MCA territory) (1); Multiple cortical infarctions and aortic arch thrombi (1)	NA	Thrombocytopenia (5); Elevated D-dimer (5)	Anti-PF4 antibodies – positive in all	No	UFH (1); Argatroban (4); Steroids (3); IVIG (3); Eculizumab (2); Alteplase (1)	Recovered (2); Recovering (3)
See I [21]	JAMA, 30 April 2021	Case series, United States	Vaccine Adverse Event Reporting System (VAERS) to the CDC and FDA database from 02 March to 21 April 2021	12	12 (100)	18 to younger than 60 years	SARS-CoV-2-PCR -negative in 10. SARS-CoV-2 antigen test - negative in 1. Not done in 1.	J&J/Janssen, all first dose	Range, 6 - 15	CVST with intracerebral haemorrhage (7); CVST (5); SAH (1); Multiple (8); DVT (3); Splanchnic vein thrombosis (2); Pulmonary embolism (3)	High BMI (6); Hypothyroidism (1); Combined oral contraceptive use (1)	Thrombocytopenia (12); Elevated D-dimer (12); Thrombophilia panel -negative (11/11)	Anti-PF4 antibodies – positive in 11/11	No	Heparin (6); Non-heparin Anticoagulant (10); IVIG (7); Steroids (3); Platelet (4)	Died (3); ICU (3); Still in hospital (2); Discharged (4)
Vayne C [22]	The New England Journal of Medicine, 19 May 2021	Case series, France	Between 19 March and 01 April 2021 with suspected VITT	9	7 (78)	Median age - 44 (range, 21-73)	NA	AstraZeneca, NA	Range, 9 - 18	CSVT (6); Multiple (6); splanchnic vein thrombosis (4); DVT 2 ischemic stroke (1); Pulmonary embolism (2); Aortic thrombosis (1); No thrombosis (1)	NA	Thrombocytopenia (8); Elevated D-dimer (7/7); Low fibrinogen levels (5)	Anti-PF4 antibodies – positive in 7	NA	NA	NA
Althaus K [23]	Haematologica, 20 May 2021	Case series, Germany	Multicenter study between 01 February and 06 April 2021	8	5 (63)	Median age -41.5 (range, 24 - 53)	NA	AstraZeneca, NA	Range, 6 - 20	CVST (3); CVST with haemorrhage (2); Multiple (3); Pulmonary embolism (3); DVT (1)	Contraception (1); No (7)	Thrombocytopenia (8); Elevated D-dimer (5/5); Low fibrinogen levels (3/5); Factor V Leiden – heterozygous (1)	Anti-PF4 antibodies – positive in all; Platelet activation assay – positive in 8/8	NA	Endovascular rheolysis (1); Anticoagulation (5); IVIG (4); Non-heparin anticoagulation (4)	Died (3)
Pomara C [24]	Haematologica, 20 May 2021	Case series, Italy	Patients admitted	2	1 (50)	50, 37	SARS-CoV-2 molecular test – negative in both	AstraZeneca, NA	10, 10	Splanchnic vein thrombosis (1); CVST with intracerebral haemorrhage (1)	No	Thrombocytopenia (2); Elevated D-dimer (2); Low fibrinogen levels (2)	Anti-PF4 antibodies – positive in all	NA	Nadroparin (1); Platelet (1); Neurosurgical intervention (1)	Died (2)
Dias L [25]	Journal of Stroke and Cerebrovascular Diseases, 25 May 2021	Case series, Portugal	Cases admitted	2	2 (100)	47, 67	SARS-CoV-2-PCR -negative in both	Pfizer/BioNTech, first dose in one patient and second dose in another patient	6, 3	CVST with SAH (1); CVST (1)	Iron-deficiency anaemia due to adenomyosis (1); Combined oral contraceptives use (1); History of multiple cerebral cavernous malformations, hypertension, diabetes, dyslipidaemia, viral myocarditis, and depression (1)	Platelet normal (2); Low protein S (1); CT - probable renal cell carcinoma (1)	Anti-PF4 antibodies – negative in both (but checked 2 months and 20 days respectively post event)	NA	Enoxaparin (2); Warfarin (1); Dabigatran (1)	Discharged (2)
Bourguignon A [26]	The New England Journal of Medicine, 09 June 2021	Brief report, Canada	First patients with thrombotic complications in Canada	3	1 (33)	63, 69, 72	NA	AstraZeneca, NA	18, 12, 7	Multiple (3); Pulmonary embolism (2); DVT (2); CVST (1); Ischemic stroke (MCA territory and ICA thrombus); (1) Splanchnic vein thrombosis (1)	Non–insulin-dependent DM, HTN, obstructive sleep apnoea and recently diagnosed prostate cancer (1)	Thrombocytopenia (3); Elevated D-dimer (3); Low fibrinogen levels (3)	Anti-PF4 antibodies – positive in all	History of heparin exposure 9 months back in one patient. No exposure in others	UFH (1); Tinzaparin (1); Fondaparinux (2); Rivaroxaban (1); Argatroban (1); PE (1); IVIG (3)	Still in hospital (1); Recovering (1); Discharged (1)
Pawlowski C [27]	Journal of Stroke and Cerebrovascular Diseases, 16 June 2021	Retrospective cohort, United States	People vaccinated in the Mayo Clinic hospital system between 01 January 2017 and 15 March 2021 and underwent at least one SARS-CoV-2 PCR test	3	2 (67)	All with CVST age ≥ 65	NA	Pfizer/BioNTech, all first dose	Within 30 days of vaccination	CVST (3)	Localized cancer (1); Heart disease (2); IBD, peptic ulcer disease, rheumatic disease, and renal disease (1)	NA	NA	NA	NA	NA
Fan BE [28]	American Journal of Hematology, 16 June 2021	Case series, Singapore	Multicenter	3	2 (67)	54, 62, 60	NA	Pfizer/BioNTech, all second dose	1, 9, 8	CVST with haemorrhage (3); SAH (2); Multiple (1); Pulmonary embolism (1); Others (1)	Dyslipidaemia (2); DM (1); HTN (2); Family history of unprovoked pulmonary embolism (1)	Platelet normal (2); Thrombophilia panel negative (2); Low anti-thrombin III (1)	Anti-PF4 antibodies – negative in 1/1; Heparin induced platelet aggregation – negative in 2/2	NA	UFH (2); LMWH (3); Warfarin (2)	Recovering (2); Recovered and discharged (1)
Esba LCA [29]	Expert Review Of Vaccines, 17 June 2021	Case series, Saudi Arabia	Hospital’s internal electronic safety reporting system from December 2020 to 13 April 2021	5	2 (40)	27, 38, 40, 61, 61	NA	AstraZeneca Pfizer/BioNTech (first dose in one patient and second in two patients, NA in others)	Both CVST - 14 Cardiac arrest - same day Pulmonary embolism - 9, 6	CVST (2); Cardiac arrest (1); Pulmonary embolism (2)	DM (2); HTN (2); COPD (1); Heart failure (1); Dyslipidaemia (1); Smoking (2); NA (1)	Thrombocytopenia (2/4)	HIT screen - negative in 1/1	NA	No treatment (1); Enoxaparin (4); Heparin (1); Apixaban (2)	Discharged (3); Died (1); Still in hospital (1)
Gattringer T [30]	Stroke & Vascular Neurology, 08 July 2021	Case series, Austria	Patients admitted	2	2 (100)	39, 24	SARS-CoV-2-PCR -negative in 1 and positive in 1	AstraZeneca, both first dose	6, 8	CVST (1); CVST with haemorrhage (1)	No (2)	Thrombocytopenia (2); Elevated D-dimer (2); Low fibrinogen levels (2); Thrombophilia panel negative (2)	Anti-PF4 antibodies – positive in both	NA	Danaparoid (1); Argatroban (2); Dabigatran (2); Steroids (2); IVIG (2)	Discharged (2)
Bano F [31]	BMJ Case Reports, 13 July 2021	Case series, United Kingdom	All patients presented over a period of 5 days to a single center	3	2 (67)	61, 53, 55	SARS-CoV-2-PCR -negative in all	AstraZeneca, all first dose	13, 11, 8	CVST with haemorrhage (2); Pulmonary embolism (1)	Asthma, HTN, high BMI, hormone replacement therapy and indapamide intake (1); Fibromyalgia (1); No (1)	Thrombocytopenia (3); Elevated D-dimer (3); Low fibrinogen levels (3)	Anti-PF4 antibodies – positive in all; Platelet functional assay - positive in 1/3	No	LMWH (1); Fondaparinux (1); Argatroban (1); Platelet (3); Cryoprecipitate (1); IVIG (1); Steroids (2); Neurosurgical intervention (1)	Recovered and discharged (1); Died (2)
Schulz JB [32]	Annals of Neurology, 19 July 2021	Retrospective survey, Germany	Web-based questionnaire emailed to neurology department of hospitals, data collection closed on 14 April 2021	59	44/58 (76)	Mean age (SD) - 46.7 (17.1); (of total 62 cerebrovascular events); Median - 46 (range, 20-89)	NA	AstraZeneca Pfizer/BioNTech (56 with first dose and 3 with second dose)	Range, 1 - 25 in CVST patients	Total cerebrovascular events (62); Total vascular events (59); CVST (45); Ischemic stroke (9); Haemorrhagic stroke (4); Others (7)	Smoking (6/52); Obese (3/59); Previous thrombosis event (1/59)	Thrombocytopenia (In 26/43 CVST and 3/9 ischemic stroke patients); Elevated D-dimer (In 23/33 CVST and 3/4 ischemic stroke patients)	Anti-PF4 antibodies – positive in 22/31 CVST patients and 5/5 ischemic stroke patients	NA	IVIG, steroids, heparin, other anticoagulants	Died (11)

Patient and Study Characteristics

Thromboembolic (venous and arterial) and hemorrhagic complications following COVID-19 vaccination were reported in 202 patients. The age group with the highest incidence was 20-40 years (39-7%; 46/116), followed by 41-60 years. The majority of patients were female (71.1%; 143/201). The mean age of presentation was 47 ± 15.5 (mean ± SD) years and ranged from 21 to 86 years.

Comorbidity and Risk Factors

Comorbidities and risk factors were documented in 122 patients, and 28 (23%) patients had significant and specific risk factors for the events; 29 (23.8%) patients had at least one medical condition prior to the onset of events. Significant risk factors included a history of deep vein thrombosis (DVT) (n = 2), a history of relapsing thrombotic thrombocytopenic purpura (TTP) (n=1), a family history of unprovoked pulmonary embolism (n = 1), previous other thrombotic events (n = 1), oral contraceptive pill use (n = 5), hormone replacement therapy (n = 3), obesity (n = 12), antiphospholipid antibodies (n = 6), Factor V Leiden mutation (n = 5), prothrombin G20210A mutation (n = 2), low antithrombin III (n = 1), heterozygous methylenetetrahydrofolate reductase (MTHFR) mutation (n = 1), low ADAMTS13 activity (n = 4), hypothyroidism (n = 4), cancer (n = 4), smoker and ex-smoker (n = 11), hypertension (n = 14), diabetes mellitus (n = 8), and atrial fibrillation (n = 2). In 72 cases, heparin exposure status was documented, and only one patient among them had a history of prior heparin exposure nine months ago; 94 (77%) of the patients had no significant risk factors for any events. The majority of studies reported that there has not been a recent COVID-19 infection and that SARS-CoV-2-PCR test results were negative.

Dosage and Vaccination

In 199 of the reported cases, information on the type of vaccine received was available. One hundred fifty-three of them received the AstraZeneca vaccine, 26 received the Pfizer/BioNTech vaccine, 16 received the J&J/Janssen vaccine, and four received the Moderna vaccine. The vaccine dose was documented in 152 patients, with 138 patients experiencing complications after the first dose (most common with the AstraZeneca vaccine) and another 14 experiencing complications after the second dose. Among the patients who developed complications with the second dose, most (11 patients) received the Pfizer/BioNTech vaccine.

Events Reported

The total number of thrombotic, thromboembolic, and hemorrhagic events reported among 202 patients was 306. Of those, 229 (74.8%) were venous thromboembolic events, and 39 (12.7%) were arterial thromboembolic events. Among them, in 174 patients for whom data was available, the mean duration of the onset of symptoms after vaccination was 9.8 ± 5 days. The majority of patients (79.9%) reported their initial symptoms between six and 14 days after vaccination, with the duration ranging from 30 minutes to 37 days. CVST was the most commonly reported form among all thromboembolic events, followed by pulmonary embolism and splanchnic thrombosis. Multiple thromboses were reported in 59 individuals (29.2%).

Nature of the Reported Events

**Figure 2:**
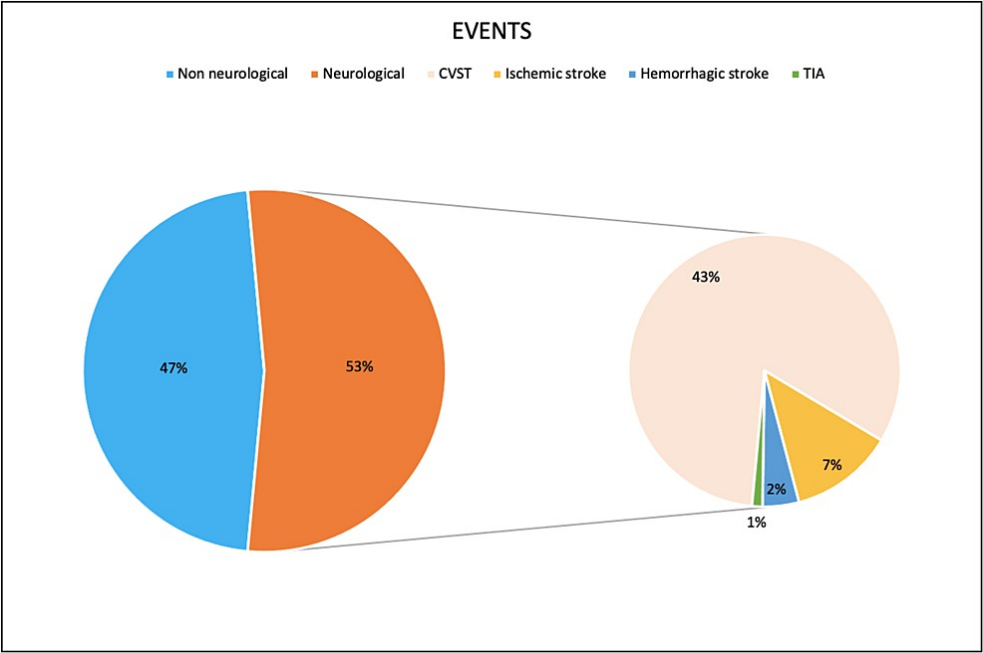
The nature of the reported thrombotic and thromboembolic events

CVST was reported in 133 patients (65.8%), and among them, 33 (24.8%) had associated brain parenchymal haemorrhage secondary to CVST. Also, 12 patients also had coexisting internal jugular vein thrombosis (IJVT). Other common systemic thromboses reported were pulmonary embolism (17.8%), splanchnic vein thrombosis (12.9%), and DVT (7.4%). Twenty patients presented with ischemic stroke, and the most common territory involved was the MCA territory (3/5 of reported cases). Other territories reported were the basilar artery and cerebellar infarcts. Two patients had transient ischemic attacks (TIAs), and one patient had an ICA non-occlusive thrombus. Seven patients had hemorrhagic strokes at admission. One patient had cardiac arrest, and three patients had myocardial infarction, including one case of triple coronary artery thrombosis and one case of right coronary artery thrombosis. Systemic venous system thromboses, such as iliac, inferior vena cava, iliofemoral, basivertebral, and bilateral superior ophthalmic vein thrombosis, were identified in seven cases. Other arterial systemic thromboses were found in 13 patients, with the most common being aortic thrombosis, followed by celiac, splenic, iliac, spinal, and femoral artery thrombosis. In five patients, cutaneous thrombosis associated with skin necrosis, dermal petechiae, and subdermal hematoma was reported. Five individuals had immune thrombocytopenic purpura (ITP), although one of them had a history and a family history of thrombocytopenia. Four patients were diagnosed with TTP. Six patients developed DIC during the course of the illness. Adrenal haemorrhage was reported in five patients. Thromboses and bleeding caused more neurological events (CVST, stroke, and TIAs) than non-neurological events (Figure 2).

Investigations

The majority of patients (78.6%) had thrombocytopenia, with the lowest platelet count reported being 1 × 10^9^/L. A large number of individuals (87.4%) had elevated D-dimer. Heparin-induced thrombocytopenia (HIT) screen/anti-PF4 antibodies were tested in 150 patients and were positive in 86% of them. Four individuals had a heterozygous Factor V Leiden mutation, two had a prothrombin G20210A mutation, and one had a methylenetetrahydrofolate reductase (MTHFR) mutation. Five individuals tested positive for lupus anticoagulant, and one patient each had low protein S and low antithrombin III levels.

Treatment and Outcome

Treatment data were available for 161 patients. The majority of patients (128; 79.5%) received heparin or non-heparin anticoagulants. Intravenous immunoglobulin (IVIG) was given to 61 patients, steroids to 42 patients, and plasmapheresis (PE) to 11 patients. Intravenous methylprednisolone was the corticosteroid most frequently used, but data were scarce. Rituximab and eculizumab were administered to four and two patients, respectively, and one patient received caplacizumab. Out of the nine patients who improved completely, five received IVIG, one received PE, seven received IVIG or PE with steroids and heparin or other non-heparin anticoagulants, and two received only anticoagulants. In four cases, neurosurgical decompression procedures were performed. In 185 patients, data on their outcomes were available. At the time of publication, 110 patients were still being treated in the hospital, four patients were critical or in ICU care, and 49 patients (26.5%) had died. Twenty-two patients were discharged, and nine of those patients had fully recovered. The rest of the patients had focal neurological deficits. Data on the cause of death was available for 35 of the 49 patients who died. 15/35 patients had thrombosis in two or more organs. In total, 26/35 patients had CVST, and 15 of them had isolated CVST. Other events reported as causes of death are pulmonary embolism (8), splanchnic vein thrombosis (7), cardiac events (3), adrenal haemorrhage (2), hemorrhagic stroke (2), and ischemic stroke (2). Out of the 27 patients who expired and whose treatment data was documented, 14 patients didn’t receive any steroids, IVIG, anticoagulants, or immunosuppressants. Although data was lacking, patients with normal D-dimer and platelet counts had better outcomes. Only six patients received IVIG, and eight patients received combination therapy. Five patients received only heparin. Data on the type of vaccine administered among those who died was available in 48 patients, and 41 (85.4%) patients received the AstraZeneca vaccine, three patients each received the Pfizer/BioNTech and J&J/Janssen vaccines, and only one received the Moderna vaccine.

National Registries, Surveillance, and Reporting Agencies' Data

According to a population-based cohort study conducted by Pottegård et al. using data from the nationwide healthcare registers of Denmark and Norway, 281264 patients received their first dosage of the AstraZeneca vaccine between February 9 and March 11, 2021. There were 59 venous thromboembolic events and 83 arterial events recorded in this vaccination group. DVT and ischemic heart disease were the most common venous thromboembolic and arterial events, respectively. The case fatality rate was 10.6% (15/142) [33]. A nested incident-matched case-control study from Scotland found that 1.71 million people received the first dose of the AstraZeneca vaccine and 0.82 million people received the Pfizer/BioNTech vaccine. The AstraZeneca vaccination was linked to 893 venous thromboembolic events and 3288 arterial thromboembolic events. There were 421 venous thromboembolic events and 1603 arterial thromboembolic events linked to the Pfizer/BioNTech vaccine. They didn't provide any information on the case fatality rate (Table 3) [34].

**Table 3:** Characteristics of studies included from nationwide registries and surveillance Abbreviations: CVST, cerebral venous sinus thrombosis; DVT, deep vein thrombosis; SAH, subarachnoid haemorrhage; ITP, immune thrombocytopenic purpura, NA, not available n=total number of patients

First Author	Published journal, date of publication	Study design, study setting	Study population	Total number of patients (n) with venous or arterial events	Sex Female, %	Age (years), mean ± SD or median (range) or median (IQR)	COVID-19 tests	Vaccine type, dose	Time period from vaccination to first symptoms (days)	Diagnosis	Comorbidities and risk factors	Blood investigations	Antiplatelet antibodies and platelet-activation assay	Previous exposure to heparin	Treatment received	Outcome
Pottegård A [33]	The BMJ, 05 May 2021	Population-based cohort study, Denmark and Norway	Nationwide healthcare registers; Denmark (n=148792); Norway (n=132472)	142	Denmark, 80.1; Norway, 77.6%	Range, 18-65, Median (IQR) age: Denmark 45 (33-55); Norway 44 (32-55)	Before vaccination, 6.2% in Denmark and 1.4% in Norway positive result for any COVID-19 tests	AstraZeneca vaccine, all first dose	NA	Total venous thromboembolic events (59); CVST (7); Pulmonary embolism (2); DVT (22) Splanchnic vein thrombosis (< 5); Arterial events (83); Ischemic heart disease (52; Intracerebral haemorrhage (8); Ischemic stroke (16); SAH (<5); ITP (<5); Bleeding events (74)	NA	Thrombocytopenia (17)	NA	NA	NA	Died (15)
Simpson CR [34]	Nature Medicine, 09 June 2021	Nested incident-matched case-control study, Scotland	National prospective COVID-19 surveillance cohort in Scotland	6205	NA	≥18 years	NA	AstraZeneca, all first dose; Pfizer/BioNTech, all first dose	NA	AstraZeneca: Venous thromboembolic events (893); Arterial thromboembolic events (3288); ITP (27); Haemorrhagic events (278); Pfizer/BioNTech: Venous thromboembolic events (421); Arterial thromboembolic events (1603); ITP (10); Haemorrhagic events (137)	NA	AstraZeneca vaccine: Thrombocytopenia (excluding ITP) (94); Pfizer/BioNTech: Thrombocytopenia (excluding ITP) (34)	NA	NA	NA	NA

As of September 1, 2021, data from the US Vaccine Adverse Event Reporting System (VAERS) reported by the CDC and FDA revealed that more than 14.3 million doses of the J&J/Janssen vaccine and more than 356 million doses of the Moderna vaccine had been administered. There were 45 events of TTS following the J&J/Janssen vaccine and two cases after the Moderna vaccine [9]. According to safety update data from the UK, 24.8 million first doses and 24.1 million second doses of the AstraZeneca vaccine were administered up to September 1, 2021, based on yellow cards reported and reviewed by the Medicines and Healthcare Products Regulatory Agency (MHRA). After the AstraZeneca vaccine, 416 cases of major thromboembolic events with concomitant thrombocytopenia were documented, with 148 of these being CVST. Overall incidence following first or unknown doses was 14.9 per million doses, and second doses were 1.9 cases per million doses. A total of 21.9 million first doses and 18.1 million second doses of Pfizer/BioNTech were administered, with 17 occurrences of similar events recorded. Only two cases of such events were recorded out of the total 1.4 million initial doses and 0.9 million second doses of the Moderna vaccine administered. The case fatality rate reported was 17% with the AstraZeneca vaccine and 12% with the Pfizer/BioNTech vaccine [10]. A case of hemorrhagic stroke (no prior comorbidities) was reported after COVAXIN vaccine administration in India (MOHFW, AEFI (adverse events following immunization) report, National AEFI Committee) (Table 4) [35].

**Table 4:** Characteristics of data from various reporting agencies Abbreviations: TTS, Thrombosis with thrombocytopenia syndrome; CVST, cerebral venous sinus thrombosis; NA, not available; MOHFW: Ministry of Health and Family Welfare; AEFI: adverse events following immunization

Reporting agency	Country	Date of issue	Type of vaccine	Total doses administered	Events reported (n)	Sex (n) and age group	Fatality
Centers for Disease Control and Prevention (CDC) and US Food and Drug Administration (FDA) Vaccine Adverse Event Reporting System (VAERS) [9]	United States	01 September 2021	J&J/Janssen and Moderna	J&J/Janssen: over 14.3 million doses; Moderna: over 356 million doses	J&J/Janssen (TTS: 45); Moderna (TTS: 2)	N/A	N/A
Medicines and Healthcare products Regulatory Agency (MHRA) [10] Yellow card scheme	United Kingdom	09 December 2020 to 01 September 2021	AstraZeneca, Pfizer/BioNTech, and Moderna	AstraZeneca: 24.8 million first doses and 24.1 million second doses; Pfizer/BioNTech: 21.9 million first doses and 18.1 million second doses; Moderna: 1.4 million first doses and 0.9 million second doses	AstraZeneca: 416 major thromboembolic events with concurrent thrombocytopenia; 45 after second dose; CVST: 148 Pfizer/BioNTech: 17 major thromboembolic events with concurrent thrombocytopenia; Moderna: two major thromboembolic events with concurrent thrombocytopenia	AstraZeneca: Female (210); male (202); unknown (94); age: range, 18-93 years; Pfizer/BioNTech: female (6) Male: 11; age: range, 28-91 years; two cases with Moderna; both male and under the age of 50	AstraZeneca: 72 deaths (17%), (6/72 after second dose); Pfizer/BioNTech: two deaths (12%); Moderna, no deaths
MOHFW, AEFI report from India [35]; National AEFI Committee	India	16 January 2021 to 28 June 2021	COVISHIELD (AstraZeneca) and COVAXIN	NA	32 patients reported with events (32); only one case with COVAXIN; 21 cardiac events; two unexplained deaths; four Ischemic stroke cases; three haemorrhagic stroke; One case of venous haemorrhagic infarct; N/A (1)	Eight females; 24 males	Seven recovered; 25 dead

Risk of Bias

In our study, 26/59 of the papers were of fair quality (NIH Quality Assessment Tool) [13]. There were 17 good-quality articles and 16 poor-quality ones (Tables 5-6).

**Table 5:** Risk of bias assessment for case series and case reports using NIH quality assessment tool Abbreviations: CD, cannot determine; NA, not applicable; NR, not reported; Y, yes; N, no

First Author	Was the study question or objective clearly stated?	Was the study population clearly and fully described, including a case definition?	Were the cases consecutive?	Were the subjects comparable?	Was the intervention clearly described?	Were the outcome measures clearly defined, valid, reliable, and implemented consistently across all study participants?	Was the length of follow-up adequate?	Were the statistical methods well-described?	Were the results well-described?	Total	Impression Case series - good quality, 6-8; fair quality, 4-5; poor quality, <4 Case reports - good quality, 5; fair quality, 3-4; poor quality, <3
Tarawneh O	N	N	NA	NA	Y	N	Y	NA	Y	3/6	Fair
Toom S	N	Y	NA	NA	Y	N	N	NA	N	2/6	Poor
Carli G	Y	N	NA	NA	Y	Y	N	NA	Y	4/6	Fair
Helms JM	Y	N	NA	NA	Y	N	N	NA	N	2/6	Poor
Franchini M	Y	Y	NA	NA	Y	N	Y	NA	Y	5/6	Good
D'Agostino V	Y	N	NA	NA	Y	N	Y	NA	Y	4/6	Fair
Muir KL	Y	Y	NA	NA	Y	Y	N	NA	Y	5/6	Good
Bayas A	Y	N	NA	NA	Y	N	N	NA	Y	3/6	Fair
Muster V	Y	N	NA	NA	Y	N	N	NA	N	2/6	Poor
Sissa C	N	Y	NA	NA	Y	N	Y	NA	N	3/6	Fair
Thaler J	Y	N	NA	NA	Y	Y	Y	NA	Y	5/6	Good
Blauenfeldt RA	Y	N	NA	NA	Y	Y	Y	NA	Y	5/6	Good
Garnier M	Y	N	NA	NA	Y	N	N	NA	Y	3/6	Fair
Bjørnstad-Tuveng TH	Y	N	NA	NA	Y	Y	Y	NA	N	4/6	Fair
Yocum A	Y	N	NA	NA	Y	Y	N	NA	Y	4/6	Fair
Xie C	N	N	NA	NA	Y	N	NR	NA	N	1/6	Poor
Tajstra M	Y	N	NA	NA	N	N	Y	NA	N	2/6	Poor
Jamme M	N	N	NA	NA	N	N	Y	NA	Y	2/6	Poor
Graf T	Y	N	NA	NA	Y	Y	NR	NA	Y	4/6	Fair
Ryan E	Y	N	NA	NA	Y	Y	N	NA	Y	4/6	Fair
Julian JA	N	Y	NA	NA	Y	N	N	NA	N	2/6	Poor
Ramdeny S	Y	N	NA	NA	N	N	N	NA	N	1/6	Poor
Zakaria Z	Y	N	NA	NA	Y	Y	N	NA	N	3/6	Fair
de Bruijn S	Y	Y	NA	NA	Y	Y	N	NA	Y	5/6	Good
Hocking J	Y	Y	NA	NA	Y	Y	N	NA	Y	5/6	Good
Al-Maqbali JS	Y	N	NA	NA	Y	N	N	NA	Y	3/6	Fair
Suresh P	Y	N	NA	NA	Y	N	Y	NA	N	3/6	Fair
Barral M	N	N	NA	NA	Y	N	N	NA	N	1/6	Poor
Tølbøll Sørensen AL	Y	Y	NA	NA	Y	Y	Y	NA	Y	6/6	Good
Aladdin Y	Y	Y	NA	NA	N	Y	Y	NA	N	4/6	Fair
Dutta A	Y	N	NA	NA	Y	N	N	NA	N	2/6	Poor
Sangli S	Y	N	NA	NA	Y	Y	Y	NA	Y	5/6	Good
Ramessur R	Y	N	NA	NA	N	N	N	NA	N	1/6	Poor
Clark RT	Y	N	NA	NA	Y	Y	Y	NA	Y	5/6	Good
Ikenberg B	Y	Y	NA	NA	Y	Y	N	NA	Y	5/6	Good
Taylor P	Y	N	NA	NA	N	Y	N	NA	N	2/6	Poor
Wang RL	Y	Y	NA	NA	Y	N	N	NA	N	3/6	Fair
Bandapaati S	Y	Y	NA	NA	Y	Y	N	NA	N	4/6	Fair
Malik B	Y	N	NA	NA	Y	Y	Y	NA	N	4/6	Fair
Waqar SHB	Y	N	NA	NA	N	N	N	NA	Y	2/6	Poor
Wolf ME	Y	Y	Y	Y	Y	Y	N	Y	Y	8/9	Good
Greinacher A	Y	N	CD	Y	Y	N	N	Y	N	4/9	Fair
Schultz NH	Y	Y	CD	Y	Y	Y	Y	NA	Y	7/9	Good
Scully M	Y	Y	CD	Y	Y	Y	CD	Y	N	6/9	Good
Mehta PR	Y	Y	CD	N	Y	Y	Y	CD	Y	6/9	Good
Haakonsen HB	Y	N	CD	Y	Y	Y	N	NA	Y	5/9	Fair
Tiede A	Y	Y	Y	Y	Y	Y	N	Y	Y	8/9	Good
See I	Y	Y	N	Y	Y	Y	N	Y	Y	7/9	Good
Vayne C	Y	N	NR	NR	Y	Y	NR	NA	NR	3/9	Poor
Althaus K	Y	N	NR	N	Y	Y	N	NA	N	3/9	Poor
Pomara C	Y	N	NR	Y	Y	N	Y	NA	Y	5/9	Fair
Dias L	Y	Y	NR	N	N	N	N	NA	Y	3/9	Poor
Bourguignon A	Y	N	Y	N	Y	Y	N	NA	Y	5/9	Fair
Fan BE	Y	N	N	Y	Y	N	N	NA	Y	4/9	Fair
Esba LCA	Y	N	Y	N	Y	Y	N	NA	N	4/9	Fair
Gattringer T	Y	Y	NR	N	Y	N	Y	NA	Y	5/9	Fair
Bano F	Y	N	Y	Y	Y	Y	Y	NA	Y	7/9	Good

**Table 6:** Risk of bias assessment for observational studies using NIH quality assessment tool Abbreviations: CD, cannot determine; NA, not applicable; NR, not reported; Y, yes; N, no

First Author	Was the research question or objective in this paper clearly stated?	Was the study population clearly specified and defined?	Was the participation rate of eligible persons at least 50%?	Were all the subjects selected or recruited from the same or similar populations (including the same time period)? Were inclusion and exclusion criteria for being in the study prespecified and applied uniformly to all participants?	Was a sample size justification, power description, or variance and effect estimates provided?	For the analyses in this paper, were the exposure(s) of interest measured prior to the outcome(s) being measured?	Was the timeframe sufficient so that one could reasonably expect to see an association between exposure and outcome if it existed?	For exposures that can vary in amount or level, did the study examine different levels of the exposure as related to the outcome (e.g., categories of exposure, or exposure measured as a continuous variable)?	Were the exposure measures (independent variables) clearly defined, valid, reliable, and implemented consistently across all study participants?	Was the exposure(s) assessed more than once over time?	Were the outcome measures (dependent variables) clearly defined, valid, reliable, and implemented consistently across all study participants?	Were the outcome assessors blinded to the exposure status of participants?	Was loss to follow-up after baseline 20% or less?	Were key potential confounding variables measured and adjusted statistically for their impact on the relationship between exposure(s) and outcome(s)?	Total	Impression Good quality, 10-14 Fair quality, 6-9 Poor quality, <6
Pawlowski C	Y	Y	Y	Y	Y	N	Y	NA	Y	NA	N	NA	Y	N	8/14	Fair
Schulz JB	Y	Y	CD	Y	Y	N	Y	NA	N	NA	N	NA	Y	N	6/14	Fair
Pottegård A	Y	Y	Y	N	N	Y	Y	NA	Y	NA	Y	N	Y	Y	9/14	Fair
Simpson CR	Y	Y	Y	Y	Y	N	Y	NA	Y	NA	N	NA	Y	N	8/14	Fair

Discussion

In this systematic review, we assessed the thrombotic and thromboembolic complications after receiving the COVID-19 vaccination, which included 59 articles with 202 patients and 306 events. Apart from that, data from two nationwide registries and surveillance revealed 6347 venous and arterial thromboembolic incidents. We also analyzed data from various reporting agencies separately. To the best of our knowledge, this is the most comprehensive review of the subject.

Data on the underlying mechanism of the AstraZeneca vaccine initiating TTS supports a two-step mechanism similar to the pathogenesis of autoimmune heparin-induced thrombocytopenia (HIT). As a result of a pronounced B-cell response triggered by vaccine-induced PF4/adenovirus aggregates and pro-inflammatory reactions, anti-PF4 antibodies are formed. These are IgG antibodies that activate platelets via low-affinity platelet FcγIIa receptors and the high-titer anti-PF4 antibodies that, in turn, stimulate platelets and neutrophils, causing neutrophils to release NETs (neutrophil extracellular traps). This leads to thrombosis in TTS [36]. The mechanism through which other types of vaccinations cause TTS is unknown. 

For a definitive diagnosis of TTS, the American Society of Hematology requires that all five points listed below be met [12] (1) administratration of the COVID vaccine four to 42 days prior to symptom onset; (2) any venous or arterial thrombosis (often cerebral or abdominal); (3) thrombocytopenia (platelet count < 150 × 10^9^/L) (4) positive PF4 HIT ELISA; (5) markedly elevated D-dimer (more than four times the upper limit of normal).

The vast majority of our patients met all five criteria, although a few did not, owing to an insufficient evaluation in many studies, such as anti-PF4 antibodies, D-dimer, etc. The WHO interim guidance case definition of TTS includes the COVID vaccine 30 days before symptom onset [37].

TTS appears to be extremely uncommon. According to CDC and FDA data, the J&J/Janssen vaccine caused 3.1 cases per million doses, and the Moderna vaccine caused 0.006 cases per million doses [9]. The MHRA in the United Kingdom reported an overall incidence of major thromboembolic events with concurrent thrombocytopenia of 8.5 cases per million doses after the AstraZeneca vaccine, but 14.9 cases per million doses after first or unknown doses, 1.9 cases per million doses after second doses, and 0.4 cases per million doses after the Pfizer/BioNTech vaccine [10]. Only two cases of TTS were reported by the CDC and FDA after more than 356 million doses of Moderna vaccine were administered in the United States, and only two cases of major thromboembolic events with concurrent thrombocytopenia were reported by the MHRA after 1.4 million first doses and 0.9 million second doses of Moderna vaccine were administered in the United Kingdom [9,10]. So the highest incidence of TTS is with AstraZeneca, followed by J&J/Janssen, and the least with the Moderna vaccine. The majority of people who received the AstraZeneca vaccine experienced complications after the first dose. The majority of those who developed complications after the second dose of the vaccine received the Pfizer/BioNTech vaccine. Other adenoviral vaccines, such as Gam-COVID-Vac/SputnikV and Ad5-nCOV, have not been linked to thromboembolic complications. It's possible that this is related to the usage of different adenovirus species serotypes or insufficient reporting [38]. As previously stated, thrombotic and thromboembolic complications were more common in our study with the AstraZeneca vaccination. However, it is too early to comment on vaccine type and predisposition to these side effects, as the majority of people received the AstraZeneca vaccine, and thus the events reported were higher with the same vaccine. More research is needed to compare the risk of thromboembolic complications among different types of vaccines.

The most common events reported were venous thromboembolic events, with CVST being the most common, followed by pulmonary embolism and splanchnic thrombosis. Compared to this, the most common venous thromboembolic events recorded in HIT are pulmonary embolism and DVT [39]. Other events reported in TTS were DVT, internal jugular vein thrombosis, ischemic stroke, TIA, hemorrhagic stroke, cardiac arrest, myocardial infarction, thrombosis in the iliac, inferior vena cava, iliofemoral, basivertebral, bilateral superior ophthalmic veins, and arterial thrombosis in the aorta, celiac, splenic, iliac, spinal, femoral, and leg arteries, cutaneous thrombosis associated with skin necrosis and dermal petechiae, subdermal hematoma, ITP, TTP, DIC, and adrenal hemorrhage. TTS, therefore, occurs more frequently in atypical regions, such as the cerebral venous sinuses and splanchnic circulation, and HIT more frequently manifests as DVT and pulmonary embolism. Furthermore, DVT and pulmonary embolism are the most often observed thrombotic complications associated with COVID-19 infection [40].

In individuals with minor symptoms or who do not require hospitalization, the reported incidence of thrombotic complications after COVID-19 was reported to be very low. The reported incidence of venous thromboembolism (VTE) was only 0.09% [41]. In a study by Hill et al., only 3.1% of 2748 patients hospitalized with COVID-19 developed VTE, including DVT (1.5%) and pulmonary embolism (1.3%), while only 0.3% had arterial thromboses [41]. Another meta-analysis found a 14.1% in-hospital prevalence of VTE (pulmonary embolism or DVT), with a greater frequency in ICU patients (22.7%) [42]. Various other studies found the pooled incidence of ischemic stroke in COVID-19 patients to be 1.2%, CVST to be 0.08%, and myocardial infarction to be 0.5% in hospitalized, non-ICU-treated patients [43]. Hence, the prevalence of thrombotic complications, notably VTE, is substantially higher after COVID-19 infection than after COVID-19 vaccination, with a much higher prevalence rate among severe COVID-19 cases. It might be attributed to longer hospital stays among COVID-19 patients who are severely ill.

The majority of patients who developed thromboembolic events were young and female. HIT, which has a similar underlying mechanism, is also more common in females than in males [44]. The majority of the patients had no prior medical history or risk factors for the events.

Most of the patients in our study had the onset of symptoms within 14 days of receiving the vaccine dose, ranging from 30 minutes to 37 days post-vaccination. A complete blood count, peripheral smear, D-dimer, fibrinogen, anti-PF4 heparin antibodies on the enzyme-linked immunosorbent assay (ELISA), and imaging (computed tomography or magnetic resonance imaging with venography or angiography) should be used to rule out thrombosis in individuals suspected of having TTS. In the absence of heparin treatment, anti-PF4 antibodies are highly indicative of TTS. Our patients showed thrombocytopenia, high D-dimer levels, and positive HIT screens or anti-PF4 antibodies in the majority of cases. The platelet count and D-dimer levels were normal in a couple of the cases. Patients with normal D-dimer and platelet counts had better outcomes, despite the dearth of available data.

A systematic review of VIIT and CVST after AstraZeneca and J&J/Janssen vaccines, which included 14 articles and 49 patients, was recently published [45]. The majority of the patients in that study were female, and the PF4 IgG assay and D-dimer were both positive in the majority of the cases. Our research yielded comparable results. Symptoms emerged in the majority of patients one week after the first vaccine dosage (range, 4-19 days) in their study. However, the majority of patients in our research developed symptoms within 14 days of receiving the vaccination dose (a range of 30 minutes to 37 days). It's possible that this is related to the fact that we incorporated more publications.

In patients with suspected TTS, a multidisciplinary approach is suggested, comprising physicians from neurology, neurosurgery, hematology, critical care, internal medicine, radiology, and the emergency department [37]. Heparin is not advised in any of the recommendations. Platelet infusion should be avoided because of its similarities to HIT, except in emergencies where surgery is strongly indicated or severe thrombocytopenia is present. IVIG and non-heparin-based anticoagulants are recommended. The recommended IVIG dose is 1 g/kg per day for two days or 0.4 g/kg per day for five days. Parenteral direct thrombin inhibitors (argatroban or bivalirudin), direct oral anticoagulants (dabigatran, rivaroxaban, apixaban), fondaparinux, and danaparoid are non-heparin anticoagulants that are recommended [12,37,46]. If IVIG treatment is ineffective, the NICE guideline also suggests adding short courses of high-dose corticosteroids (methylprednisolone 1 g for three days or dexamethasone 20 to 40 mg for four days) [46]. Aspirin should be avoided because it can increase the risk of bleeding and is ineffective in preventing TTS [12]. Plasma exchange, eculizumab, and rituximab can be considered as treatment options in patients not responding to IVIG and anticoagulation [12,46]. Only a small number of individuals received these treatments (plasma exchange, eculizumab, and rituximab), and the prognosis was good. Those who received IVIG had a better prognosis as compared to others.

The limitation of our study was that the majority of the research in our review was case reports. The likelihood of thromboembolic complications reported following COVID-19 vaccination may be greater due to the potential underreporting of side effects. The strength of our analysis is that we used data from national registries, monitoring agencies, and reporting agencies, as well as observational studies and case reports, to compile it. The majority of the studies were of fair to good quality.

## Conclusions

COVID-19 vaccines are generally safe, and the risk of death from COVID-19 disease far outweighs the risk of TTS from the COVID-19 vaccine. Thrombotic complications are more likely due to COVID-19 infection than post-vaccination, so concern about these events should not deter patients from getting the COVID-19 vaccine. Clinicians should be aware of TTS as it is potentially fatal, and early diagnosis and appropriate treatment can save lives. Vaccine side-effect profiles must be evaluated in future trials.
